# Model-Based Approach for Optimizing Ceftobiprole Dosage in Pediatric Patients

**DOI:** 10.1128/AAC.01206-21

**Published:** 2021-10-18

**Authors:** Christopher M. Rubino, Anthony P. Cammarata, Anne Smits, Sebastian Schröpf, Mark Polak, Karine Litherland, Kamal Hamed

**Affiliations:** a Institute for Clinical Pharmacodynamics, Inc., Schenectady, New York, USA; b Neonatal Intensive Care Unit, University Hospitals Leuven, Leuven, Belgium; c Department of Development and Regeneration, KU Leuven, Leuven, Belgium; d Dr. von Hauner Children’s Hospital, Ludwig-Maximilians-University Munich, Munich, Germany; e West Virginia University School of Medicine, Morgantown, West Virginia, USA; f Basilea Pharmaceutica International Ltd., Basel, Switzerland

**Keywords:** ceftobiprole, cephalosporin, pediatric patients, pharmacokinetics, population pharmacokinetics

## Abstract

Ceftobiprole is an advanced-generation cephalosporin for intravenous administration with activity against Gram-positive and Gram-negative organisms. A population pharmacokinetic (PK) model characterizing the disposition of ceftobiprole in plasma using data from patients in three pediatric studies was developed. Model-based simulations were subsequently performed to assist in dose optimization for the treatment of pediatric patients with hospital-acquired or community-acquired pneumonia. The population PK data set comprised 518 ceftobiprole plasma concentrations from 107 patients from 0 (birth) to 17 years of age. Ceftobiprole PK was well described by a three-compartment model with linear elimination. Ceftobiprole clearance was modeled as a function of glomerular filtration rate; other PK parameters were scaled to body weight. The final population PK model provided a robust and reliable description of the PK of ceftobiprole in the pediatric study population. Model-based simulations using the final model suggested that a ceftobiprole dose of 15 mg/kg of body weight infused over 2 h and administered every 12 h in neonates and infants <3 months of age or every 8 h in older pediatric patients would result in a ceftobiprole exposure consistent with that in adults and good pharmacokinetic-pharmacodynamic target attainment. The dose should be reduced to 10 mg/kg every 12 h in neonates and infants <3 months of age who weigh <4 kg to avoid high exposures. Extended intervals and reduced doses may be required for pediatric patients older than 3 months of age with renal impairment.

## INTRODUCTION

Ceftobiprole, the active moiety of the prodrug ceftobiprole medocaril, is an advanced-generation cephalosporin for intravenous (i.v.) administration, with broad-spectrum activity against Gram-positive and Gram-negative organisms, including methicillin-resistant Staphylococcus aureus (MRSA), vancomycin-resistant S. aureus, penicillin-resistant Streptococcus pneumoniae, Enterococcus faecalis, and Pseudomonas aeruginosa (1–3). It is approved in many European and other countries for the treatment of hospital-acquired pneumonia (HAP), excluding ventilator-associated pneumonia, and community-acquired pneumonia (CAP) (4). Ceftobiprole is currently under phase 3 investigation in adults to support a New Drug Application in the United States for acute bacterial skin and skin structure infections and S. aureus bacteremia (5, 6). Ceftobiprole has low protein binding (16% in adults), undergoes minimal metabolism, and is eliminated predominantly via renal excretion as unchanged drug (4).

Given its spectrum of activity, ceftobiprole represents an important potential addition to the therapeutic armamentarium for pediatric infections. To date, three clinical studies have been completed in pediatric patients: (i) Study CSI-1006, a phase 1, single-dose, pharmacokinetics (PK) and safety study in pediatric patients 3 months to <18 years of age (7); (ii) Study BPR-PIP-001, a phase 1, single-dose, PK and safety study in neonates and infants up to 3 months of age (8); and (iii) Study BPR-PIP-002, a phase 3 study in pediatric patients 3 months to <18 years of age with HAP or CAP (9). While all three studies provided important information on the PK of ceftobiprole in pediatric patients when evaluated individually, pooling of the data from these studies allowed for the application of modeling and simulation approaches that have been shown to be critical in the development of safe and effective therapies in this population (10–13). Optimal dosing regimens for pediatric therapeutics must balance both efficacy and safety. For antibacterials, it is possible to leverage pharmacokinetic-pharmacodynamic (PK-PD) targets for effective therapy to optimize dosing regimens for pediatric infections (14–16). From a safety perspective, one could bridge to the experience in adults by selecting dosing regimens that result in an overall drug exposure similar to that observed in adults receiving doses shown to be safe in large, phase 3 clinical trials (12, 13, 17).

The PK-PD index for ceftobiprole has been identified as the percentage of time that free-drug concentrations remain above the MIC of an infecting pathogen (%*fT*_>MIC_) (18). Craig and Andes characterized the PK-PD of ceftobiprole using murine infection models and showed that the %*T*_>MIC_ required to achieve net bacterial stasis was longer for Gram-negative pathogens (36 to 45% of the dosing interval) compared with Gram-positive pathogens (14 to 28%). At a MIC value of 4 μg/ml, >90% of adults with HAP enrolled in a phase 3 study, which showed that ceftobiprole was noninferior to ceftazidime plus linezolid, achieved these PK-PD targets (19). This MIC was chosen because it is the ceftobiprole non-species-specific PK-PD breakpoint as determined by The European Committee on Antimicrobial Susceptibility Testing (20). It is important to note that using a MIC of 4 μg/ml is considered conservative, given that the species-specific ceftobiprole breakpoints are ≤0.25 μg/ml for *Enterobacterales*, ≤2 μg/ml for S. aureus, and ≤0.5 μg/ml for S. pneumoniae (20).

The PK data from Studies CSI-1006, BPR-PIP-001, and BPR-PIP-002 were used to establish a pediatric population PK model for ceftobiprole, and the analyses described herein were designed to support the optimal dosing regimens of ceftobiprole for pediatric patients with HAP or CAP.

## RESULTS

### Pharmacokinetic data description.

A total of 606 plasma samples from 112 patients were available from the three studies. Sixteen quantifiable and 72 nonquantifiable samples were excluded. Three quantifiable samples from Study CSI-1006 were deemed outliers based on a conditional weighted residual (CWRES) of >3; one of these samples was obtained from a patient who had only one evaluable sample, and therefore that patient was excluded from the analysis. The remaining 13 excluded samples with quantifiable ceftobiprole concentrations were from four patients in Study BPR-PIP-002 whose data were excluded from the analysis for the following reasons: two patients had contamination of samples due to the samples being drawn from the infusion line, and two patients had insufficient sample volume for the majority of samples. Of the 72 nonquantifiable samples that were excluded, 70 were from Study CSI-1006 (i.e., 15.6% of samples from this study) and included 51 samples below the limit of quantitation (BLQ) and 19 records that had missing values in the source data. The remaining two nonquantifiable samples were BLQ samples from Study BPR-PIP-001. The resultant population PK data set thus comprised 518 ceftobiprole plasma concentrations obtained from 107 patients.

Summary statistics of baseline patient characteristics for the PK analysis population stratified by study and pooled across all studies are provided in Table 1. Patients were predominantly white (71.0%) and male (57.0%). Age ranged from 0 to 17 years (0.3 to 934.9 weeks), body weight ranged from 2.5 to 75.3 kg, and body mass index ranged from 10.0 to 38.8 kg/m^2^. A total of 14 patients from Studies CSI-1006 and BPR-PIP-002 received the maximum dose of 500 mg; no patients from Study BPR-PIP-001 received the maximum dose.

**TABLE 1:** Summary statistics of baseline patient characteristics for the population PK analysis population^*a*^

Variable	Result for:			
	CSI-1006 (*n* = 63)	BPR-PIP-001 (*n* = 15)	BPR-PIP-002 (*n* = 29)	Total (*n* = 107)
Median (min–max)				
Age				
yr	5.00 (0–17.0)	0.0220 (0.00500–0.178)	5.00 (1.00–17.0)	4.00 (0–17.0)
mo	70.3 (3.25–215)	0.263 (0.0660–2.14)	60.0 (12.0–204)	58.0 (0.0660–215)
wk				
Postnatal	306 (14.1–935)	1.14 (0.285–9.26)	260 (52.0–884)	252 (0.285–935)
Gestational	—^*b*^	39.4 (37.6–41.4)	40.0 (40.0–40.0)	40.0 (37.6–41.4)
Postconceptional	—^*b*^	42.0 (38.4–48.5)	300 (92.0–924)	248 (38.4–924)
ht, cm	118 (55.9–189)	54.0 (49.0–61.0)	116 (83.0–180)	109 (49.0–189)
wt, kg	21.4 (5.60–75.3)	3.98 (2.50–5.27)	23.0 (11.0–67.0)	18.5 (2.50–75.3)
BMI, kg/m^2^	18.1 (11.0–38.8)	13.8 (10.0–15.6)	16.2 (13.6–23.3)	17.2 (10.0–38.8)
BSA, m^2^	0.850 (0.280–1.95)	0.230 (0.180–0.290)	0.850 (0.510–1.81)	0.740 (0.180–1.95)
GFR_Rhodin, FFM_ (ml/min)	64.3 (11.5–144)	5.90 (3.83–10.4)	58.9 (38.2–134)	52.3 (3.83–144)

*n* (%)				
Gender				
Male	35 (55.6)	10 (66.7)	16 (55.2)	61 (57.0)
Female	28 (44.4)	5 (33.3)	13 (44.8)	46 (43.0)
Race				
White	34 (54.0)	13 (86.7)	29 (100)	76 (71.0)
Black	23 (36.5)	1 (6.67)	0 (0)	24 (22.4)
Other/unspecified	6 (9.52)	1 (6.67)	0 (0)	7 (6.54)

a Abbreviations: BMI, body mass index; BSA, body surface area; GFR, glomerular filtration rate; max, maximum; min, minimum; Rhodin_FFM_, Rhodin formula based on fat-free mass.

b —, gestational and postconceptional age not reported for Study CSI-1006.

**FIG 1:**
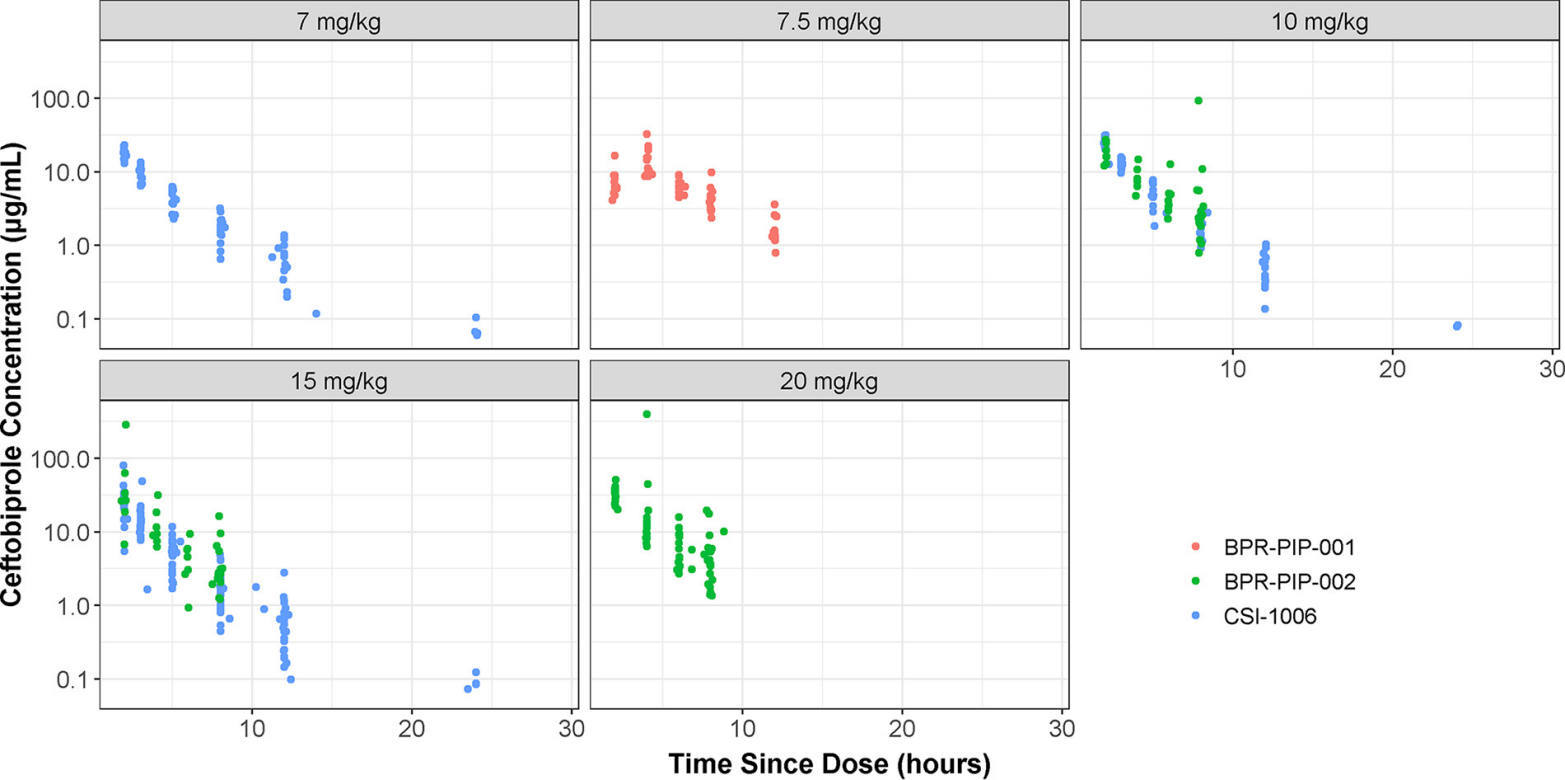
Semilog scatterplots of plasma concentrations versus time, stratified by study and presented by dose.

Consistent with the respective protocols, the majority of patients (81 of 107) provided five PK samples each; the median number of samples per patient was five, and the range was 1 to 6 (one patient had a single sample and two patients had two samples each). Plots of the observed ceftobiprole concentrations over time are provided in Fig. 1. The PK samples were spread throughout the 12- to 24-h sampling windows as expected. Concentrations appeared to increase with increasing dose. The minimal amount of accumulation with repeated dosing every 8 h (q8h) was apparent in the 10- and 15-mg/kg groups as the concentrations observed in patients enrolled in Study BPR-PIP-002, who provided PK samples on day 3, were only slightly higher than those observed in patients from Study CSI-1006, who received single doses.

### Population pharmacokinetic modeling.

Model development was initiated using a previous model that had been developed using the data from patients enrolled in Study CSI-1006 and an initial cohort of six patients enrolled in Study BPR-PIP-001. While the previous structural model provided a reasonable fit to the pooled data from all three pediatric studies, the fitted estimate for nonrenal clearance (CL_NR_) was quite low (0.0242 liter/h, which was 10-fold lower than the estimate of 0.277 liter/h from the previous model) and the shrinkage in the interindividual variability (IIV) estimate for volume of distribution for peripheral compartment 1 (*V_p_*_1_) was high (104%). For these reasons, several permutations of the model were attempted prior to proceeding to the covariate screening analysis. Ultimately, the following revisions were made to construct the final base model: (i) fixing of the CL_NR_ to zero as the models in which it was estimated or fixed to the previous value of 0.277 liter/h resulted in poor fits to the data or were overparameterized (based on the condition number), (ii) estimation of IIV on volume of distribution for the central compartment (*V*_1_) only (i.e., neither *V_p_*_1_ nor volume of distribution for peripheral compartment 2 [*V_p_*_2_]), and estimation of the weight-based scaling factor for the volume terms.

**FIG 2:**
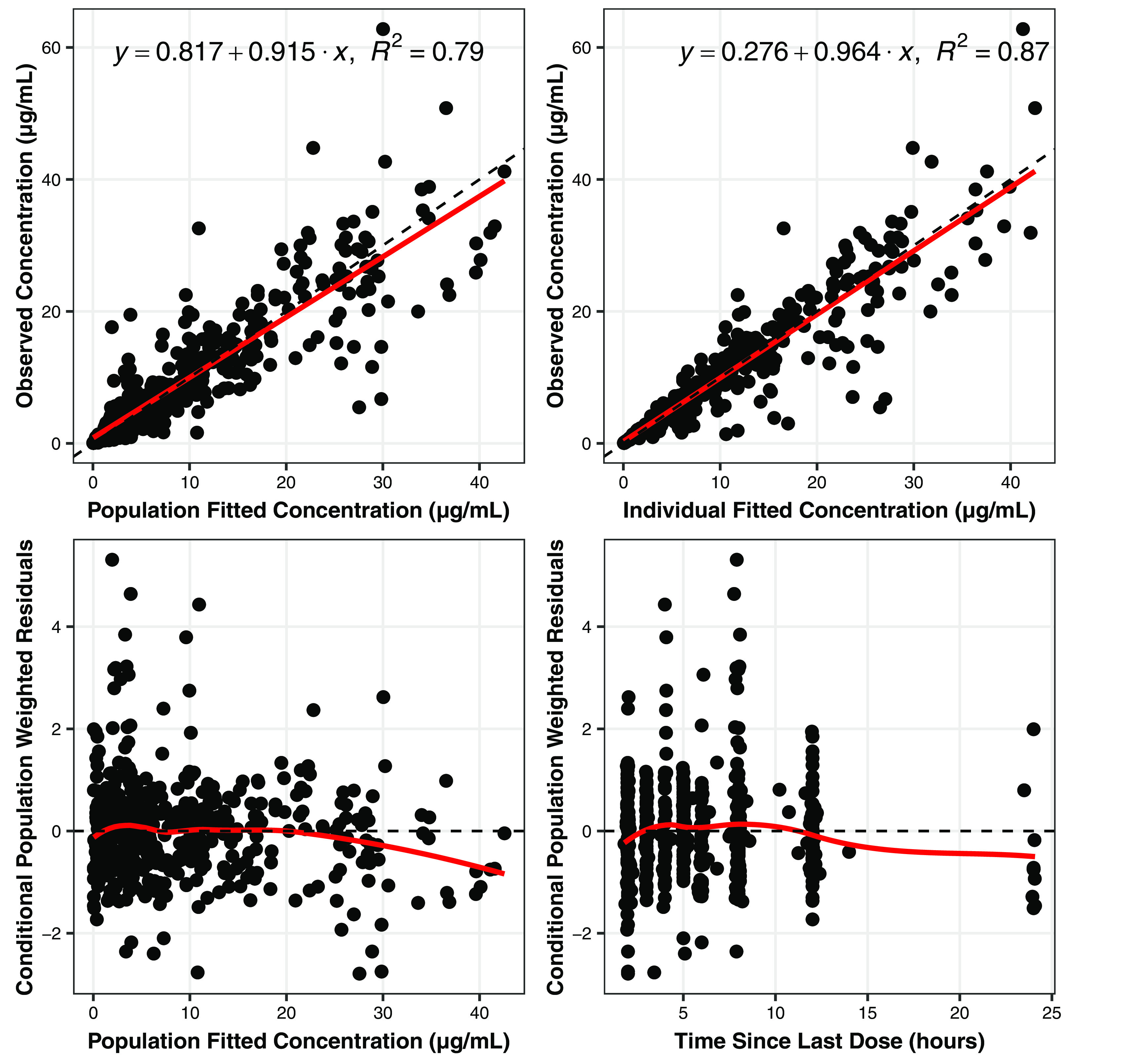
Goodness-of-fit plots for the fit of the final model to the pooled data set.

The goodness-of-fit plots for the final base model are provided in Fig. 2. Although the improvement was modest relative to the previous model, the adequacy of the fit and the associated 12-unit drop in the minimum value of the objective function (MVOF) indicated that this model was preferred. The overall distribution of normalized prediction distribution errors (NPDE) appeared to be symmetrical around a value of zero and did not appear to deviate from a normal distribution, suggesting that the model fitted the data with minimal bias. The histograms of NPDE are available in Fig. S1 in the supplemental material.

The base model described above was utilized to generate plots of the IIV in clearance (CL) and *V*_1_ versus the patient characteristics of interest. The covariate screening plots failed to reveal any additional relationships between patient descriptors and primary PK parameters. The covariate screening plots are provided in Fig. S2 to S4 in the supplemental material. Thus, further covariate model development (i.e., forward selection, full multivariable model refinement, and backward elimination) was not conducted. The final base model was therefore declared as the final population PK model and was subjected to the model qualification analyses.

The final population PK model was a three-compartment model with linear elimination. Interindividual variability was estimated for CL and *V*_1_. Residual variability was described using an additive plus proportional error model. Ceftobiprole CL was modeled as a function of glomerular filtration rate (GFR) estimated using the method of Rhodin based on fat-free mass (GFR_Rhodin,FFM_) (21). The volume terms (*V*_1_, *V_p_*_1_, and *V_p_*_2_) were scaled to body weight using a fitted coefficient, while the distributional clearance terms (distributional clearance from *V*_1_ to *V_p_*_1_ [*Q*_1_] and distributional clearance from *V*_1_ to *V_p_*_2_ [*Q*_2_]) were scaled to body weight using a coefficient fixed to 0.75, consistent with allometric scaling principles (22).

The population PK parameter estimates and associated standard errors for the model are provided in Table 2, along with the resample statistics from the sampling-importance-resampling (SIR) analysis. The resampled parameter means were aligned with those estimated in the final model fit, with 95% confidence intervals (CIs) consistent with the precision of the final model. This suggests that the parameters were estimated reliably and with adequate precision. There was a modest IIV as the IIV estimates for CL and *V*_1_ were 23.4% and 26.7%, respectively. The shrinkage in the IIV estimate for CL was low (9.56%) and somewhat higher but acceptable for *V*_1_ (38.4%). Overall, the residual variability (RV) in plasma was moderate (26.5% for the proportional term and a standard deviation [SD] of 0.0125 for the additive term).

**TABLE 2:** Summary statistics of resampled population PK parameters in comparison to the model parameter estimates from the final population pharmacokinetic model^*a*^

Parameter	Final model		SIR statistics			
	Final estimate	% SEM	Mean	Median	% CV	90% CI
CL-GFR_slope_ (liters/h/ml/min)^*b*^	0.0548	4.93	0.0551	0.0552	4.48	[0.0508, 0.0587]
*V*_1_ intercept (liters)	16.1	6.14	16.1	16.0	9.65	[13.6, 18.8]
*Q*_1_ (liters/h)^*c*^	0.545	45.8	0.542	0.512	35.4	[0.282, 0.894]
*V_p_*_1_ (liters)	49.5	104	57.3	41.9	86.9	[7.05, 163]
*Q*_2_ (liters/h)^*c*^	3.46	13.4	3.66	3.58	25.1	[2.34, 5.33]
*V_p_*_2_ (liters)	6.13	7.76	6.20	6.19	13.3	[4.92, 7.66]
Vol scaling factor^*d*^	0.911	3.88	0.911	0.912	3.48	[0.86, 0.964]
ω^2^_CL_	0.0547 (% CV, 23.4)	19.4	0.0567	0.0554	19.7	[0.0405, 0.077]
ω^2^*_V_*_1_	0.0711 (% CV, 26.7)	58.4	0.0789	0.0745	38.6	[0.0372, 0.137]
σ^2^_Proportional_	0.0701 (% CV, 26.5)	15.9	0.0716	0.0715	8.27	[0.0622, 0.0817]
σ^2^_Additive_	0.000156 (SD, 0.0125)					

a Abbreviations: CL-GFR_slope_, slope term defining the relationship between GFR and CL; ω^2^_CL_, interindividual variability on clearance; ω^2^*_V_*_1_, interindividual variability on *V*_1_; σ^2^_Proportional_, proportional residual variability; σ^2^_Additive_, additive residual variability; %SEM, percent standard error of the mean; SIR, sampling-importance-resampling; *V*_1_, volume of distribution for the central compartment; *V_p_*_1_, volume of distribution for peripheral compartment 1; *V_p_*_2_, volume of distribution for peripheral compartment 2.

b Population mean CL = CL-GFR_slope_ × GFR_Rhodin,FFM_.

c Intercompartmental clearances were scaled using a factor (power coefficient) of 0.75.

d Scaling factor (power coefficient) for *V*_1_, *V_p_*_1_, and *V_p_*_2_.

The primary goodness-of-fit plots for the final population PK model demonstrated the adequacy of the model fit in this population of infected pediatric patients (Fig. 2). The NPDE plots (Fig. S1) indicated little to no bias in the fit across the range of times since last (i.e., previous) dose and by study. The model also provided a robust fit to the observed data when evaluated on a per-patient basis.

**FIG 3:**
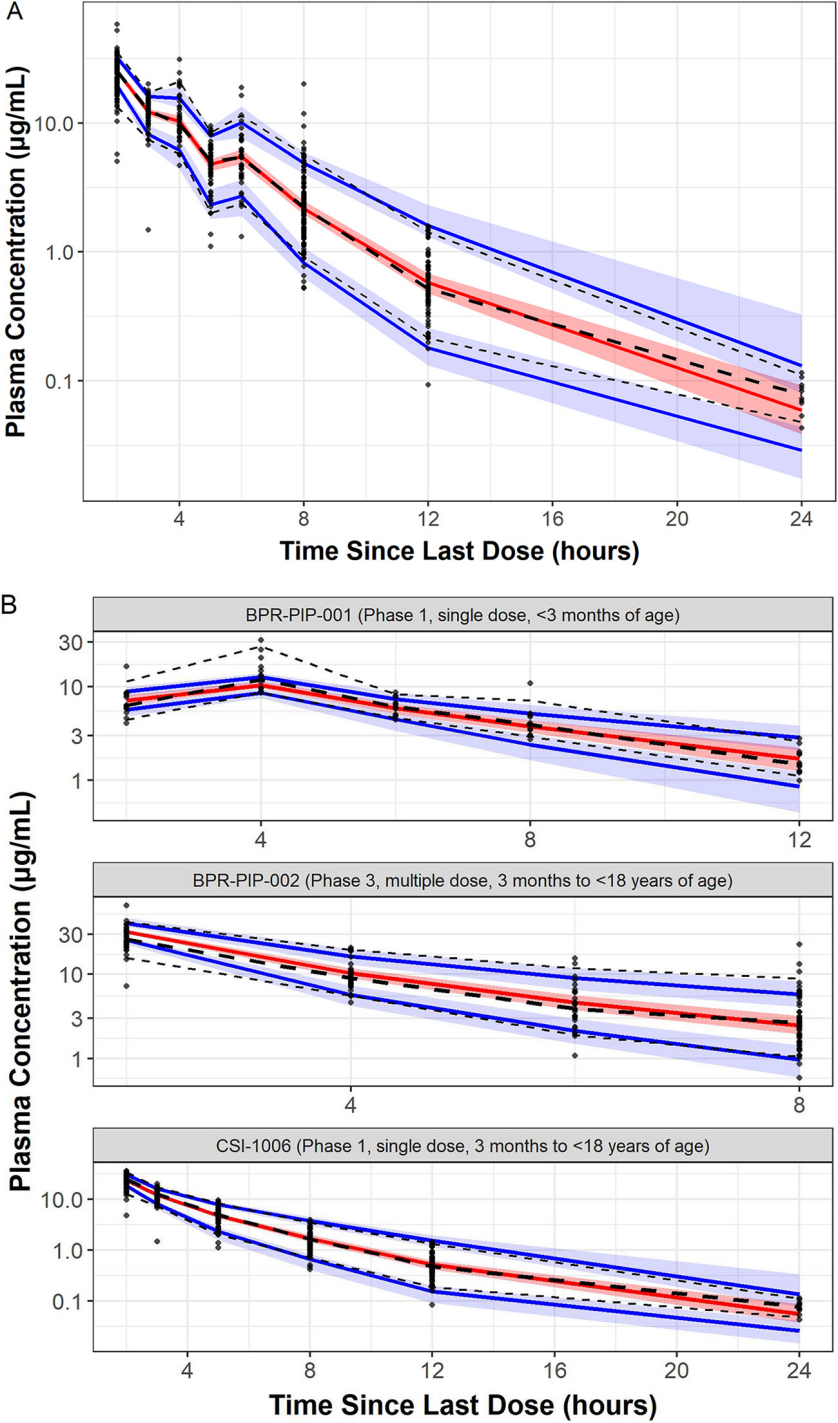
Closed circles are observed concentrations, thicker black dashed lines are the median observed concentrations, and thinner black dashed lines are the 5th and 95th percentiles of the observed concentrations. Red and blue lines and shaded regions are the 90% confidence intervals for the median and 5th and 95th percentiles from the simulations, respectively. (A) Prediction-corrected visual predictive check plot for the final model (pooled analysis data set). (B) Prediction-corrected visual predictive check plot for the final model (by study).

The prediction-corrected visual predictive check (PC-VPC) plots, constructed to provide an internal qualification evaluation, are provided for the pooled data set and stratified by study in Fig. 3. In general, the median and 5th and 95th percentiles of the observed data fell within the 90% prediction interval for the respective values from the model-based simulations. The one exception to this observation was the PC-VPC plot for Study BPR-PIP-001 data alone, which suggested that the observed concentrations at the end of the ceftobiprole infusion (i.e., at 4 h) were higher than the model predictions. This was not considered to be a deficiency in the model and did not warrant further exploration because (i) the blood volume in this newborn population was small, and therefore, issues of appropriate “mixing” could cause observed concentrations to be higher than expected immediately after the end of an infusion of this duration, and (ii) the summary statistics for the observed concentrations were based on a small number of patients (*n* = 15) and therefore were highly influenced by extreme values. Overall, the PC-VPC plots indicated that model-based simulations using the final population PK model adequately captured both the central tendency and the variability in the observed data.

In summary, the final population PK model was expected to provide robust and reliable estimates of ceftobiprole plasma exposure in the pediatric patients enrolled in Studies CSI-1006, BPR-PIP-001, and BPR-PIP-002. The simulation-based diagnostics also suggested that the model was appropriate for the conduct of model-based simulations designed to identify appropriate ceftobiprole dosing regimens in children.

### Pharmacokinetic exposure estimates and key secondary pharmacokinetic parameters.

Summary statistics for the key PK exposure parameters (maximum plasma drug concentration [*C*_max_], area under the concentration-time curve from time zero to 8 h [AUC_0–8_]), CL, and steady-state volume of distribution [*V*_ss_]) are provided in Table 3, stratified by study and dose group. When comparing patients from Studies BPR-PIP-002 and CSI-1006 who received similar doses (i.e., 10- and 15-mg/kg dose groups), the levels of exposure to ceftobiprole were similar, despite the fact that patients in Study BPR-PIP-002 received multiple doses of ceftobiprole, and the exposures therefore represent steady state as opposed to a single dose. This observation supports the relative lack of accumulation with multiple dosing due to the relatively short half-life of ceftobiprole (4). When comparing the weight-normalized CL estimates across studies, the impact of reduced renal function in the newborns is apparent as the patients enrolled in Study BPR-PIP-001 had substantially lower CL than patients from the other two studies. The weight-normalized *V*_ss_ estimates were relatively similar across doses and studies. The impact of the infusion duration was observed when comparing the *C*_max_ in patients from Study BPR-PIP-001 who received a single dose of 7.5 mg/kg as a 4-h infusion to that in patients from Study CSI-1006 who received a slightly lower dose of 7 mg/kg as a 2-h infusion; the *C*_max_ was lower in patients from Study BPR-PIP-001 (median of 11.1 μg/ml) than that in patients from Study CSI-1006 (median of 17.1 μg/ml).

**TABLE 3:** Summary of key ceftobiprole PK parameters in infected pediatric patients, derived from the fit of the population PK model, stratified by study and dose regimen^*a*^

Parameter	Mean (% CV); median (min–max)						
	BPR-PIP-001 (7.5 mg/kg [*n* = 15])^*b*^	BPR-PIP-002^*b*^^,^^*c*^			CSI-1006^*b*^		
		10 mg/kg (*n* = 8)	15 mg/kg (*n* = 6)	20 mg/kg (*n* = 15)	7 mg/kg (*n* = 16)	10 mg/kg (*n* = 15)	15 mg/kg (*n* = 32)
*C*_max_ (μg/ml)	11.6 (17.0); 11.1 (9.26–16.7)	22.8 (9.45); 22.7 (19.4–26.3)	30.3 (21.3); 29.1 (23.2–42.5)	37.5 (9.27); 36.7 (31.2–44.5)	17.0 (8.37); 17.1 (14.8–19.7)	22.8 (7.92); 22.7 (19.5–26.0)	27.6 (8.54); 27.1 (23.4–33.3)
AUC_0–8_ (μg · h/ml)	50.0 (18.4); 49.1 (37.5–67.6)	63.8 (22.1); 59.6 (50.6–93.1)	76.7 (30.1); 71.6 (53.7–120)	104 (23.7); 93.2 (74.6–159)	48.8 (15.2); 50.8 (35.1–58.8)	59.4 (13.0); 58.1 (45.6–72.4)	71.3 (17.1); 71.5 (51.4–102)
CL (liters/h/kg)	0.0834 (22.2); 0.0810 (0.056–0.109)	0.116 (27.2); 0.117 (0.073–0.154)	0.144 (26.1); 0.152 (0.091–0.187)	0.157 (21.4); 0.160 (0.097–0.214)	0.113 (19.8); 0.106 (0.088–0.160)	0.133 (14.5); 0.134 (0.107–0.175)	0.171 (19.8); 0.164 (0.109–0.242)
*V*_ss_ (liters/kg)	1.31 (6.13); 1.33 (1.16–1.43)	1.07 (4.26); 1.06 (1.01–1.13)	1.11 (5.17); 1.11 (1.03–1.19)	1.22 (5.22); 1.20 (1.09–1.33)	1.04 (2.85); 1.03 (0.997–1.09)	1.08 (3.85); 1.08 (1.02–1.15)	1.20 (4.23); 1.20 (1.11–1.33)

a Abbreviations: AUC_0–8_, area under the plasma concentration-time curve from time zero to 8 h; CL, clearance; *C*_max_, maximum plasma drug concentration; *V*_ss_, steady-state volume of distribution.

b The durations of infusion were 4 h in Study BPR-PIP-001 and 2 h in the other two studies.

c *C*_max_ and AUC_0–8_ estimates for patients from Study BPR-PIP-002 were derived using predicted profiles after the morning dose on day 3.

### Monte Carlo simulations.

The characteristics of the simulated pediatric population are provided in Fig. S5 in the supplemental material. As expected, both patient body weight and GFR increased with increasing age. GFR (in units of ml/min) increased to adult values at an age of approximately 12 years.

Given that the exposures in the age groups 3 months to <2 years and 2 to <6 years for the initial dosing regimen were predicted to be high relative to the observed distribution in adults, several alternative regimens were attempted. Through this process, it was determined that a regimen using 15 mg/kg infused over 2 h was appropriate for these age groups. Based upon the lower CL in infants from birth to <3 months of age, it was determined that an interval of 12 h was appropriate for the youngest age cohort and an interval of 8 h was appropriate for the remaining age groups. Further evaluation of the distributions of exposure within each age group indicated that a dose reduction was warranted in infants <3 months of age with lower body weight. Allowing for a lower dose (10 mg/kg) in patients from birth to <3 months of age who weigh below 4 kg resulted in a more consistent distribution of exposures in the lowest age group. These optimized dosing regimens are described in Table 4.

**TABLE 4:** Optimal age-based dosing regimen derived from model-based simulations^*a*^

Age group	Dosing regimen by level of renal function:		
	Normal or mild impairment (GFR of ≥50 ml/min/1.73 m^2^)	Moderate impairment (GFR of 30 to <50 ml/min/1.73 m^2^)	Severe impairment (GFR of 10 to <30 ml/min/1.73 m^2^)
Birth to <3 mo	15 mg/kg q12h^*b*^	15 mg/kg q12h^*b*^	15 mg/kg q24h^*b*^
3 mo to <2 yr	15 mg/kg q8h	10 mg/kg q12h	10 mg/kg q24h
2 to <6 yr	15 mg/kg q8h	10 mg/kg q12h	10 mg/kg q24h
6 to <12 yr	15 mg/kg q8h	10 mg/kg q12h	10 mg/kg q24h
12 to <18 yr	15 mg/kg q8h	7.5 mg/kg q12h	7.5 mg/kg q24h

a Abbreviations: GFR, glomerular filtration rate; q8h, every 8 h; q12h, every 12 h; q24h, every 24 h. All regimens were administered as a 2-h infusion with a maximum allowable dose of 500 mg regardless of patient’s body weight.

b Patients with a body weight of <4 kg were given 10 mg/kg instead of 15 mg/kg.

**FIG 4:**
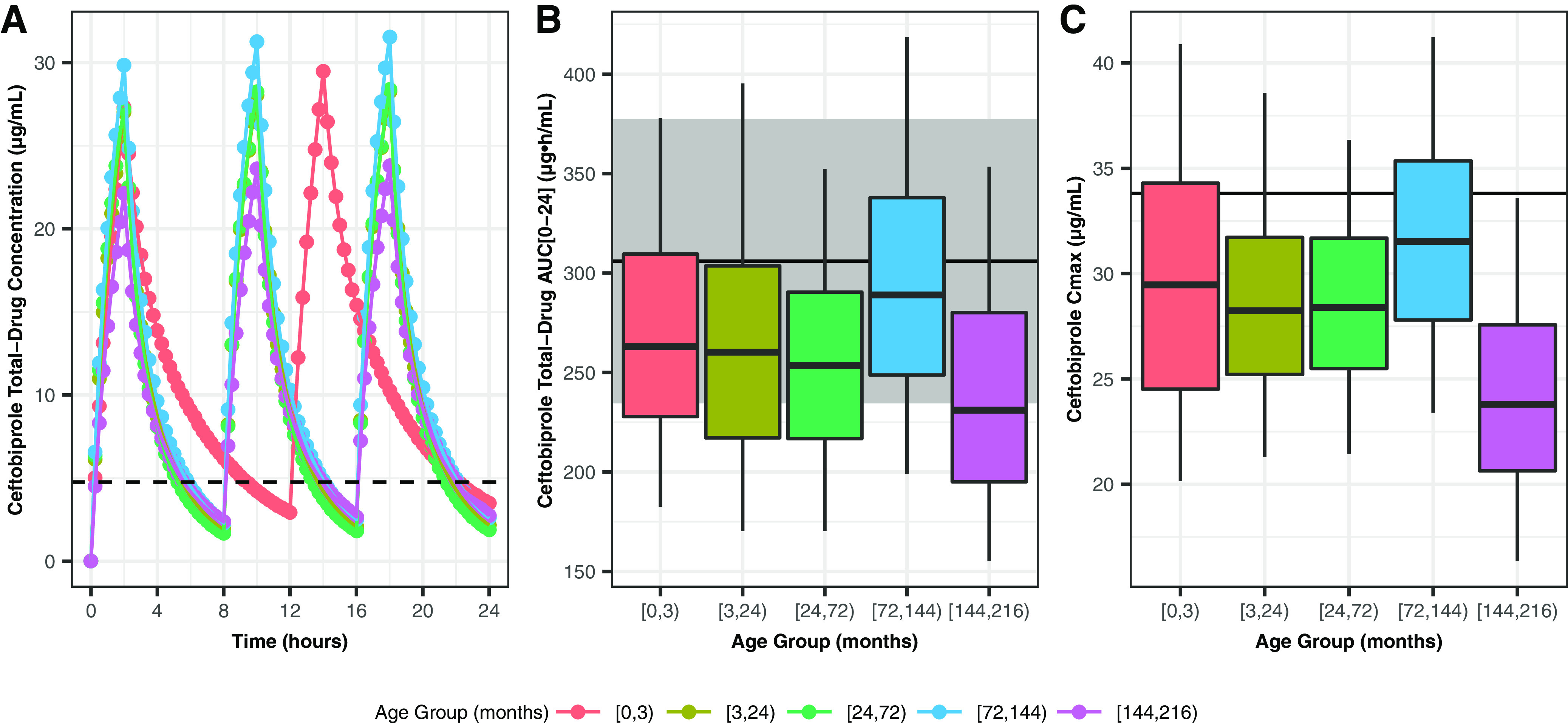
(A) Median predicted ceftobiprole plasma concentration-time profiles by age group for the optimized dosing regimen (with body weight adjustment). The dashed line corresponds to a free drug concentration of 4 μg/ml (total drug concentration of 4.76 μg/ml, assuming protein binding of 16%). (B) Distributions of predicted ceftobiprole AUC_0–24_ by age group for the optimized dosing regimen (with body weight adjustment). The black line and shaded region are the mean ± 2 standard deviations for adults from CSI-1004 (500 mg over 2 h q8h). Box-and-whisker plots show median, 25th to 75th percentiles, and 5th to 95th percentiles. (C) Distributions of predicted ceftobiprole *C*_max_ by age group for the optimized dosing regimen (with body weight adjustment). The black line and shaded region are the mean ± 2 standard deviations for adults from CSI-1004 (500 mg over 2 h q8h). Box-and-whisker plots show median, 25th to 75th percentiles, and 5th to 95th percentiles.

The median predicted ceftobiprole concentration-time profiles, stratified by age group using the optimized dosing regimen described above, are provided in Fig. 4A, which shows relatively consistent concentration-time profiles across the different age groups. The oldest age group exhibited slightly lower concentrations due to the majority of patients receiving the maximum allowable dose of 500 mg q8h. Panels B and C in Fig. 4 present the distributions of predicted area under the concentration-time curve from time 0 to 24 h (AUC_0–24_) and *C*_max_ values, respectively, for the optimized dose regimen. Summary statistics for the predicted exposures are provided in Table S1 in the supplemental material. Overall, the predicted exposures were slightly lower than those observed in the adult comparator group. However, the predicted target attainment remained adequate (Table 5).

**TABLE 5:** Predicted PK-PD target attainment by age group for the optimized dosing regimen (with body weight adjustment) derived from model-based simulations^*a*^

Target %*fT*_>MIC_	Predicted PK-PD attainment				
	Birth to <3 mo (15 mg/kg q12h)^*b*^	15 mg/kg q8h			
		3 mo to <2 yr	2 to <6 yr	6 to <12 yr	12 to 17 yr
30	100	100	100	100	100
40	99.6	97.4	97.1	98.9	98.5
50	95.8	85.4	83.6	92.9	89.4
60	86.2	66.9	61.1	79.5	70.0

a Abbreviations: %*fT*_>MIC_, percentage of the dosing interval that free-drug concentrations are above the MIC; q8h, every 8 h; q12h, every 12 h. All regimens were administered as a 2-h infusion with a maximum allowable dose of 500 mg regardless of patient’s body weight.

b Patients with a body weight of <4 kg were given 10 mg/kg q12h as a 2-h infusion.

As expected, given the relationship between renal function and ceftobiprole CL, the model-based simulations indicated that dose adjustments would be warranted in pediatric patients with renal impairment. The dosing regimens that would be predicted to result in exposures that are consistent with the adult distributions with adequate PK-PD target attainment are provided in Table 4. The predicted ceftobiprole exposures are provided in Fig. S6 and S7 in the supplemental material. Note that patients in the lowest age group (birth to <3 months) without renal impairment are expected to have normalized GFR values in the range of 30 to <50 ml/min/1.73 m^2^ (Fig. S5) and therefore do not require a dosage adjustment. Overall, despite resulting in slightly lower exposure than was observed in adults, the adjusted regimens are expected to provide adequate PK-PD target attainment for infected pediatric patients with renal impairment (see Table S2 in the supplemental material).

## DISCUSSION

These analyses had two main objectives: (i) to construct a population PK model that would describe the PK of ceftobiprole in pediatric patients with a broad range of ages from birth to <18 years and (ii) to leverage the population PK model to conduct model-based simulations to determine optimal ceftobiprole dosing regimens in pediatric patients. The PK in patients enrolled in the three pediatric studies used for the development of the population PK model herein had previously been evaluated using noncompartmental methods (7, 8). While those analyses provided important information regarding ceftobiprole exposure in the individual patients at the specific doses used in the studies, model-independent methods are limited in terms of evaluating alternative dosing regimens that may be more appropriate for clinical use. In contrast, the use of population PK methods not only allows for a robust description of the PK in the individual subjects and the identification of factors associated with PK variability but also facilitates model-based simulations that are critical to the identification of optimal dosing regimens from a PK-PD standpoint.

For this pediatric population, a three-compartment population PK model with linear elimination provided the most robust fit to the data. The IIV in CL was related to GFR as estimated by the method of Rhodin using free-fat mass (21), such that CL decreased as GFR_Rhodin,FFM_ decreased. This is consistent with the fact that ceftobiprole is predominantly eliminated by renal excretion (3, 4). It is important to note that GFR_Rhodin,FFM_ is used to estimate renal function in pediatric patients and reflects the ontogeny of renal maturation (i.e., the youngest patients have the lowest GFR_Rhodin,FFM_ in units of ml/min). Thus, the inclusion of GFR_Rhodin,FFM_ in the population PK model considers both the maturation of renal function and the changes in body size in this population. Body weight alone described adequately the IIV in the remaining PK parameters included in the model. Allometric scaling principles were invoked for the relationship between body weight and the distributional clearance terms (*Q*_1_ and *Q*_2_) in the model (i.e., using a coefficient fixed to a value of 0.75) (22). In contrast, it was feasible to fit the coefficient describing the relationship between body weight and the volume terms (*V*_1_, *V_p_*_1_, and *V_p_*_2_) in the model. The value of that fitted coefficient was 0.911, which is in line with a value of 1.0 that is often employed when using allometric scaling theory. Despite this similarity, the model in which the coefficient for the volume terms was estimated provided a statistically significant improvement in the overall model fit (data not shown). After the inclusion of body weight and renal function in the model, no other covariates were found to be predictive of the variability in ceftobiprole PK.

Specific model qualification procedures were defined *a priori* to confirm both the reliability of the estimated parameters in the population PK model (using a SIR procedure) and the ability of model-based simulations to capture the observed data (using PC-VPC plots). Overall, the results of the SIR procedure indicate that the final population PK model provided robust and reliable estimates of ceftobiprole plasma PK parameters and exposures in the patients enrolled in the pediatric studies. In addition, the PC-VPC plots illustrate the ability of model-based simulations to adequately capture the observed data, indicating that the model is appropriate for the conduct of model-based simulations designed to identify appropriate ceftobiprole dosing regimens in pediatric patients.

Model-based simulation analyses to inform pediatric dosing regimens require not only a robust model from which to extrapolate but also a structured approach to constructing a hypothetical population of subjects whose characteristics accurately reflect the complex relationship between age, sex, body size, and, for ceftobiprole, renal function. Ultimately, a ceftobiprole dose of 15 mg/kg infused over 2 h and administered every 12 h (q12h) in neonates and infants <3 months or q8h in older pediatric patients was predicted to result in ceftobiprole exposures that were consistent both across the various age groups and with those observed in adults given the approved clinical dose (23). This regimen also provided adequate PK-PD target attainment, as ≥97.1% of the pediatric population were predicted to achieve a %*fT*_>MIC_ above 40%. Further evaluation of the exposure in the youngest age group (neonates and infants <3 months) indicated that there was a tendency for increased exposure in simulated subjects weighing <4 kg. Thus, the ceftobiprole dose should be reduced from 15 mg/kg q12h to 10 mg/kg q12h in neonates and infants <3 months who weigh <4 kg to avoid excessively high exposures. There are two aspects of the PK-PD target attainment analyses that warrant consideration. First, protein binding of ceftobiprole in the pediatric population was kept identical to the protein binding estimate for adults (16%) and was used in the analyses (24). This was based on the fact that the protein binding of ceftobiprole in adults is relatively low and independent of drug concentration, as well as the lack of available neonatal and pediatric protein binding data. However, we are aware that for some other antibiotics, higher unbound drug fractions in neonates and/or children are observed compared to adults (25–29). The lack of measured unbound concentrations is a study limitation. Second, the conclusions are primarily based on the %*fT*_>MIC_ target of 40% instead of the more stringent target of 60% that is often cited for β-lactam treatment of vulnerable patients (30). While a %*fT*_>MIC_ target of 60 to 65% of the dosing interval has been identified in mouse infection models as the target of near-maximal bacterial killing (19), a target of 40% is appropriate when using a MIC of 4 μg/ml, which is considered conservative given that the species-specific ceftobiprole breakpoints are ≤0.25 μg/ml for *Enterobacterales*, ≤2 μg/ml for S. aureus, and ≤0.5 μg/ml for S. pneumoniae (20).

Simulations designed to inform appropriate dosing in pediatric patients with renal impairment indicated that extended intervals and lower doses may be required to achieve appropriate ceftobiprole exposures. It is important to note that the patients enrolled in the pediatric studies all had normal renal function for their age. Therefore, the results of the model-based simulations to inform dosing in pediatric patients with renal impairment represent an extrapolation outside the observed data. While this approach has been used to identify dosing regimens for such patients for other β-lactams that are similar to ceftobiprole in terms of the extent of renal elimination (31), the appropriateness of these regimens may warrant further investigation.

The results presented illustrate the value of pharmacometric approaches in pediatric drug development. The qualified population PK model provides an accurate and precise quantification of the disposition of ceftobiprole in pediatric patients and presents important details regarding patient characteristics that influence the IIV in ceftobiprole PK. Using model-based simulations, relatively simple dosing regimens have been proposed that are likely to result in exposures that are associated with efficacy while minimizing the potential for toxicity by maintaining exposures at or below those seen in adults receiving the approved clinical dose of ceftobiprole. By bridging to data from animal models of infection and safety information from adults, pharmacometric approaches such as these provide an efficient means for justification of dosages in pediatric patients. In this way, lengthy and logistically difficult clinical trials could be avoided, increasing the speed with which potentially life-saving therapies are available for pediatric patients.

## MATERIALS AND METHODS

All studies included in the analysis were reviewed by each study site’s Independent Ethics Committee or Institutional Review Board. Written informed consent was obtained from the parent(s) or other legally authorized representatives of the pediatric patients.

### Study CSI-1006.

Study CSI-1006 (NCT01026636) was a multicenter, open-label, single-dose, phase 1 study conducted to evaluate the PK and safety of ceftobiprole in hospitalized and ambulatory pediatric patients who had documented or presumed bacterial infections, or were at risk for them, and were undergoing treatment with systemic antibiotics (7, 32). A total of 64 patients 3 months to <18 years of age were enrolled and administered a single dose of i.v. ceftobiprole over 2 h. Patients were dosed according to age group: infants 3 months to <2 years of age received 15 mg/kg, children 2 to <6 years of age received 15 mg/kg, children 6 to <12 years of age received 10 mg/kg, and adolescents 12 to <18 years of age received 7 mg/kg. Doses were capped at a maximum of 500 mg.

Blood samples for PK analysis were collected predose and at 2, 3, 5, 8, 12, and 24 h after the start of infusion.

### Study BPR-PIP-001.

Study BPR-PIP-001 (NCT02527681) was a multicenter, open-label, single-dose, phase 1 study conducted to evaluate the PK and safety of ceftobiprole in neonates and infants up to 3 months of age undergoing treatment with systemic antibiotics (8, 33). A total of 45 patients, stratified for gestational age (GA) and postnatal age, were planned to be enrolled in three sequential cohorts: (i) full-term infants (GA of ≥37 weeks), (ii) infants with a GA of 33 to 36 weeks, and (iii) infants with a GA of 28 to 32 weeks. Due to slow enrollment, the study was completed after enrollment of the full-term cohort (*n* = 15), with no preterm patients enrolled. All patients received i.v. ceftobiprole 7.5 mg/kg as a single dose infused over 4 h. This dose was selected based on observations from Study CSI-1006 (7). Blood samples for PK analysis were obtained predose and at 2, 4, 6, 8, and 12 h after the start of dosing.

### Study BPR-PIP-002.

Study BPR-PIP-002 (NCT03439124) was a multicenter, randomized, investigator-blind, active-controlled, phase 3 study to evaluate the safety, tolerability, pharmacokinetics, and efficacy of ceftobiprole versus i.v. standard-of-care cephalosporin treatment with or without vancomycin in pediatric patients 3 months to <18 years of age with HAP or CAP requiring hospitalization (9, 34). A total of 138 patients were enrolled in the study and randomized in a 2:1 ratio to ceftobiprole (*n* = 94) or a standard-of-care comparator (*n* = 44). Seventy patients were under 6 years of age, and 68 patients were 6 years or older. Patients randomized to ceftobiprole received a ceftobiprole dose based on age. Infants 3 months to <2 years of age received ceftobiprole at 20 mg/kg over 4 h. Children 2 to <6 years of age received ceftobiprole at 20 mg/kg over 2 h, while children 6 to <12 years of age received ceftobiprole at 15 mg/kg over 2 h. Adolescents 12 to <18 years of age received ceftobiprole at 10 mg/kg over 2 h. Doses were infused q8h, and each dose was capped at 500 mg. After 3 days of i.v. therapy, patients could have been switched to an oral standard-of-care antibiotic to complete a minimum of 7 days and a maximum of 14 days of total antibiotic therapy.

Blood samples for PK analysis were obtained on day 3 based on age. For patients ≥2 years of age, samples were collected predose and at 2 (end of infusion), 4, 6, and 8 h after start of infusion. For patients <2 years of age, samples were collected predose and at 4 (end of infusion), 6, and 8 h after start of infusion.

### Bioanalytical assay.

Plasma was analyzed for total concentrations of ceftobiprole, ceftobiprole medocaril, and the open-ring metabolite using a validated gradient reversed-phase liquid chromatography-tandem mass spectrometry (23, 35).

### Previous population PK model.

Previously, a pediatric population PK model for ceftobiprole was constructed using data from patients enrolled in Study CSI-1006 and an initial cohort of six patients from Study BPR-PIP-001. A key aspect of the analysis was to identify which estimate of renal function provided the most robust prediction of ceftobiprole CL. The four approaches for estimating renal function in the pediatric population that were tested comprised traditional creatinine clearance calculation using the Schwartz formula (36), GFR estimated using the Rhodin formula based on normalized fat mass (21), GFR estimated using the Rhodin formula based on fat-free mass (i.e., GFR_Rhodin,FFM_) (21), and renal maturation function using the method of De Cock et al. (37).

Ultimately, a robust fit to the data from pediatric patients from birth to 17 years of age was achieved using a linear three-compartment model with a fitted CL_NR_ and a direct relationship between renal clearance and GFR_Rhodin,FFM_. The volume terms (*V*_1_, *V_p_*_1_, and *V_p_*_2_) were scaled to body weight using a fitted power coefficient, while the distributional clearance terms (*Q*_1_ and *Q*_2_) were scaled to body weight using a fixed allometric coefficient of 0.75. This model served as the starting point for the development of the ceftobiprole pediatric population PK model described herein.

### Population PK model development overview.

The population PK analysis was conducted using NONMEM software version 7.4, implementing the first-order conditional estimation method with interaction (38). Candidate population PK models were minimally assessed using the following criteria: evaluation of individual and population mean PK parameter estimates and their precision as measured by the percentage of standard error of the population mean estimate; graphical examination of standard diagnostic and population analysis goodness-of-fit plots (with possible stratification by various factors such as study, dose group, single-dose and multiple-dose data, etc.); graphical examination of the agreement between the observed, population-predicted, and individual *post hoc* predicted concentration-time data (individual observed and predicted overlays); reduction in both RV and IIV; comparison of the MVOF for nested models, or Akaike’s information criterion for nonnested models (39); and biologic plausibility of the parameter estimates.

Base model development was initiated using the prior model described above. Given that the previous model was developed using a subset of the data included in the analyses described herein (i.e., the patients from Study CSI-1006 and one cohort of patients from Study BPR-PIP-001), the fit of this model to the pooled data set from all three pediatric studies was assessed initially. Base model development then proceeded by investigating the potential for changes to the model to more robustly fit the pooled data. These potential modifications included the following: estimation of the IIV in the parameters for which it had not been estimated previously (*V*_1_, *Q*_1_, and/or *Q*_2_), estimation or fixing of the scaling factor used to define the relationship between the volume parameters (*V*_1_, *V_p_*_1_, and *V_p_*_2_) and body weight, and estimation (or fixing, as necessary) of CL_NR_.

Throughout development of the base model, IIV was modeled for each PK parameter where appropriate using an exponential error model assuming these parameters were log-normally distributed. A combined additive plus proportional error model was used to describe residual variability. Modifications to the RV model were only evaluated, if necessary, based upon the fit of the model to the pooled data.

The patient descriptors that were evaluated as potential covariates of PK variability included age, gestational age, postconceptional age, body weight, body mass index, sex, and race. For potential covariates recorded at the screening visit, such as age, sex, race, and body weight, it was assumed that these data remained constant over the duration of the study. Using the final base model, individual *post hoc* PK parameters were obtained for each patient. Plots of these individual *post hoc* parameter estimates minus the population mean value of the parameter were examined for observable trends. The covariate was to proceed to forward selection if there were any potential relationships identified in the covariate screen. Covariates contributing at least a 3.84-unit change in the MVOF (α = 0.05, 1 degree of freedom) were to be considered statistically significant during forward selection. After completion of forward selection, the IIV models were to be reevaluated. Finally, backward elimination was to occur if more than two covariates were included in the full multivariable model.

The final population PK model for this analysis was qualified by performing a PC-VPC (40). The PC-VPC plots were generated using PERL-speaks NONMEM for the simulations and the “vpc” package for R developed by Ron Keizer to generate the images (http://vpc.ronkeizer.com/). A SIR procedure was then conducted to assess the proposed final model’s parameter precision (41, 42). Further model refinement was undertaken if the PC-VPC plots indicated substantial bias or if the SIR procedure showed substantial differences between the parameters from the final population PK model and the corresponding estimates from the SIR procedure.

Individual predicted concentration-time profiles for each patient were generated from the Bayesian PK parameter estimates obtained from the final population PK model. This was accomplished by transitioning the NONMEM model code to C++ code so that simulations could be conducted using mrgsolve, a package for R that facilitates simulations from differential equation-based models (43).

### Monte Carlo simulations.

A data set of 5,000 hypothetical pediatric patients with appropriate body size and renal function was created. Age was simulated to approximate a uniform distribution of the following age groups: birth to <3 months, 3 months to <2 years, 2 to <6 years, 6 to <12 years, and 12 to <18 years. Approximately 50% of patients were assigned a sex of male, with the remainder being female. Data from the United States Centers for Disease Control and Prevention were used to generate height and weight values for each simulated subject based on the age and sex to which they were assigned. GFR for the simulated patients was assigned using a standard equation that assumes normal renal function, adjusted for age and body surface area (BSA) using fat-free mass (21).

Using the population PK model and the data set of patient characteristics, concentration-time profiles were simulated for each hypothetical patient for several dosing regimens using R and the mrgsolve package. Simulated concentrations were generated every 15 min for 24 h following the dose using the final population PK model. Only day 1 concentrations were simulated due to lack of clinically relevant accumulation. AUC_0–24_ was utilized for comparison purposes to account for the potential for the dosing interval to be altered in certain age groups. For each simulated patient, the %*fT*_>MIC_ was calculated using a MIC of 4 μg/ml and correcting the simulated total-drug concentration-time profiles for the protein binding of ceftobiprole (free fraction of 0.84) (1). The %*fT*_>MIC_ therefore represented the percentage of the 24-h period on day 1 during which free drug remained above 4 μg/ml.

The appropriateness of each regimen for use in pediatric patients with CAP/HAP was then evaluated based on PK-PD target attainment (for efficacy) and against a range of exposures observed in adults (for safety). For PK-PD target attainment, the goal was to select regimens that resulted in ≥90% of simulated patients achieving a %*fT*_>MIC_ of 40% at a MIC of 4 μg/ml (18). From a safety perspective, the predicted exposures were compared to the distribution of steady-state AUC and *C*_max_ estimates observed in normal healthy adults receiving ceftobiprole at 500 mg q8h for 5 days (4).

The initial dosing regimen employed for the simulations was derived from the regimens described by Bosheva et al. (9). If the initial regimen was deemed suboptimal, alternative regimens were tested to identify the regimen that provided adequate target attainment while maintaining predicted ceftobiprole exposure in the range of those observed in adults.

In addition, model-based simulations were conducted to evaluate potential dose adjustments for infected pediatric patients with renal impairment. The process involved modifying the data set of patient characteristics created by adjusting the GFR based on an expectation of moderate or severe renal impairment. A lower limit of 10 ml/min/1.73 m^2^ was chosen for the severe renal impairment simulations to limit the potential for bias when extrapolating from the model, which would predict a ceftobiprole CL of 0 at a GFR of 0. The normalized GFR was back-transformed to GFR in ml/min using the BSA for each simulated patient.
