# Supplementary material for: Model-Based Approach for Optimizing Ceftobiprole Dosage in Pediatric Patients
Source: Antimicrob Agents Chemother. 2021 Oct 18;65(11):e01206-21. doi: 10.1128/AAC.01206-21 (PMC8522765; doi:10.1128/AAC.01206-21)
Supplement: Supplemental file 1 — Supplemental material. Download AAC.01206-21-s0001.pdf, PDF file, 0.8 MB [file aac.01206-21-s0001.pdf]

Figure S1. Histograms of NPDE stratified by study/cohort (top) and overall (bottom)

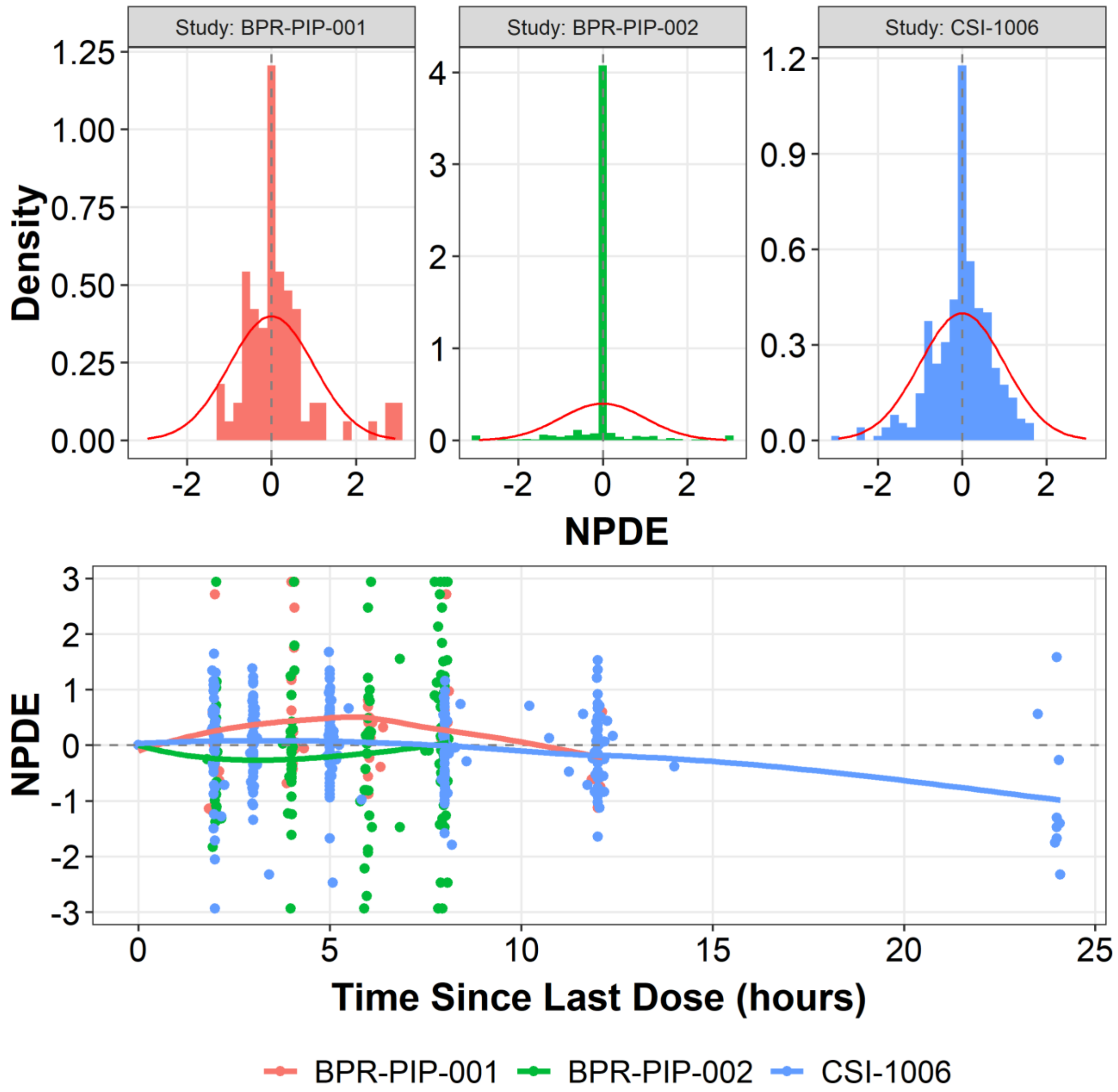

Note: NPDE, normalized prediction distribution errors. Overlain red curves in top panel represent the standard normal distribution.

Figure S2. Covariate screening plots using final base model applied to the pooled dataset – ETA_{CL} vs. continuous covariates

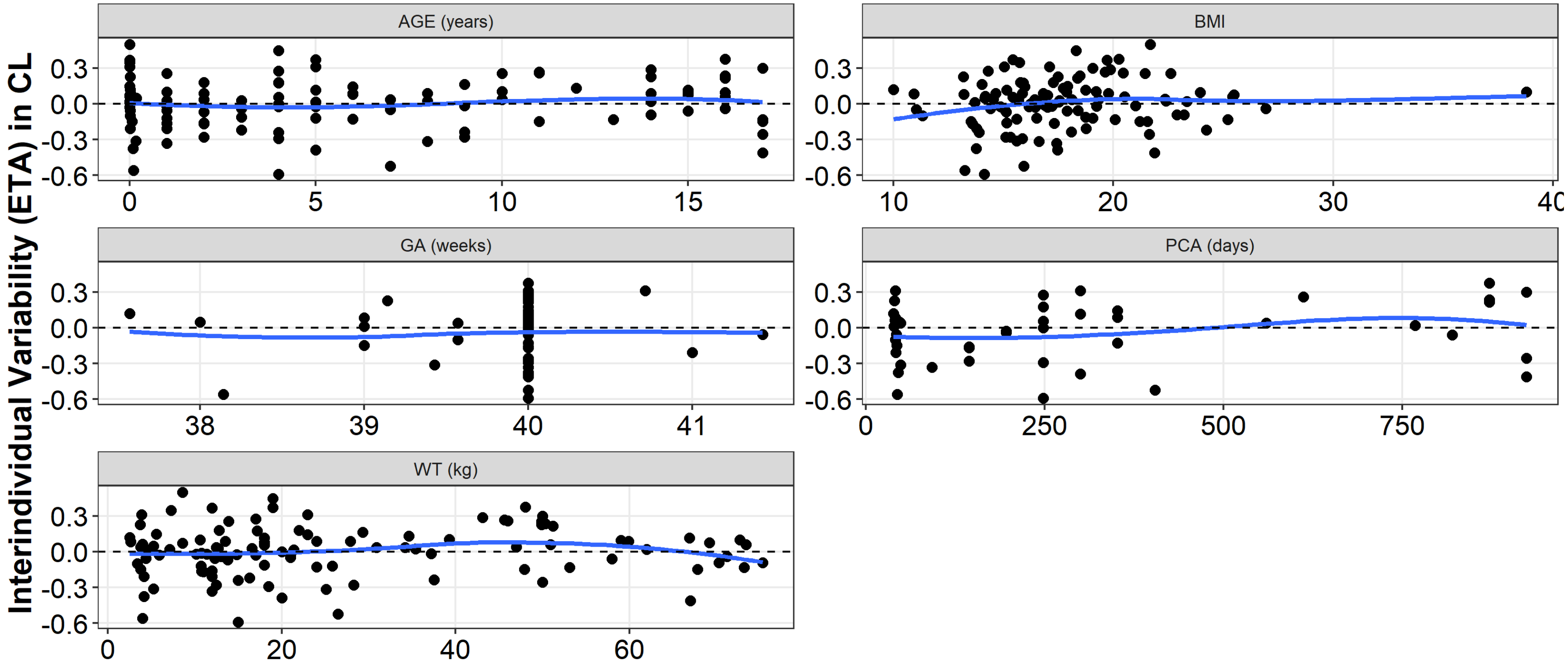

Note: BMI, body mass index; ETA_{CL}, patient-specific interindividual variability estimate in Clearance; GA, gestational age; PCA, postconceptional age; WT, weight.

Figure S3. Covariate screening plots using final base model applied to the pooled dataset – ETA_{Vc} vs. continuous covariates

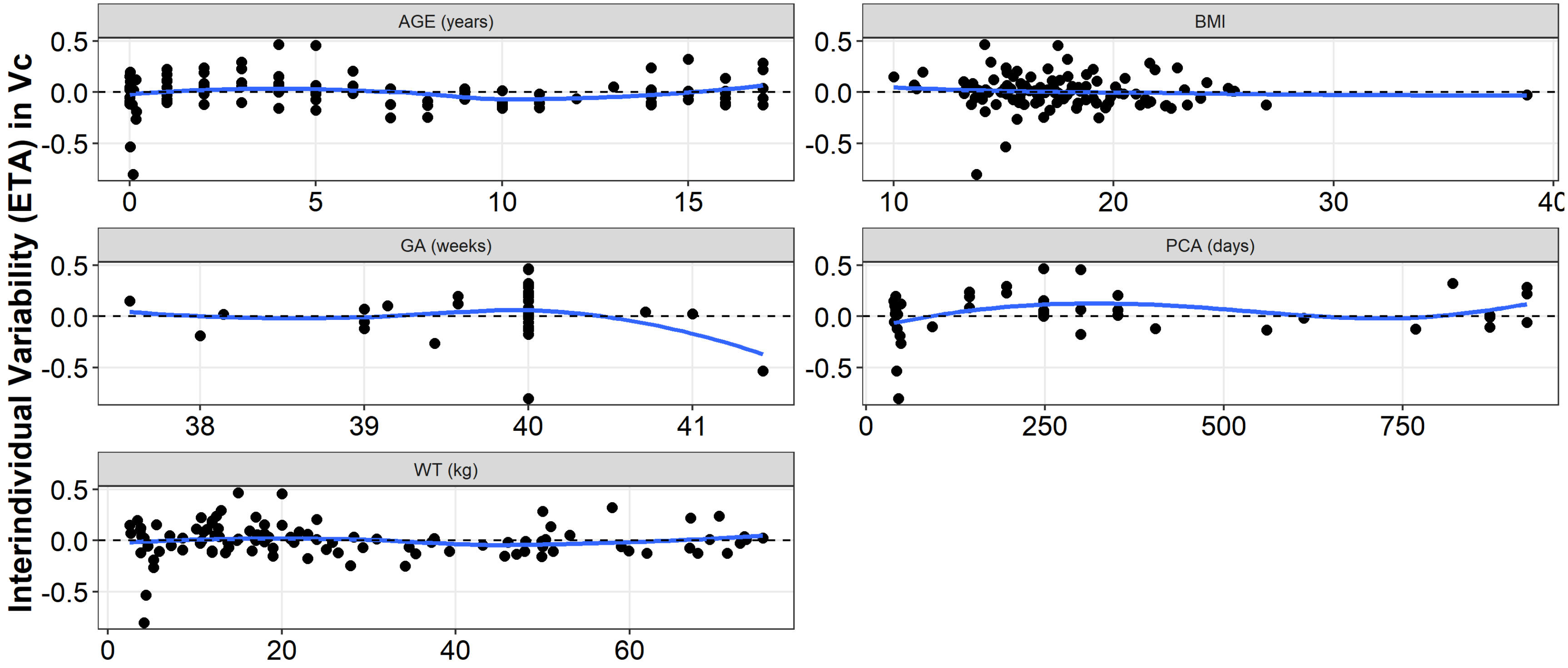

Note: BMI, body mass index; ETA_{Vc}, patient-specific interindividual variability estimate in Volume of the Central Compartment; GA, gestational age; PCA, postconceptional age; WT, weight

Figure S4. Covariate screening plots using final base model applied to the pooled dataset – categorical covariates

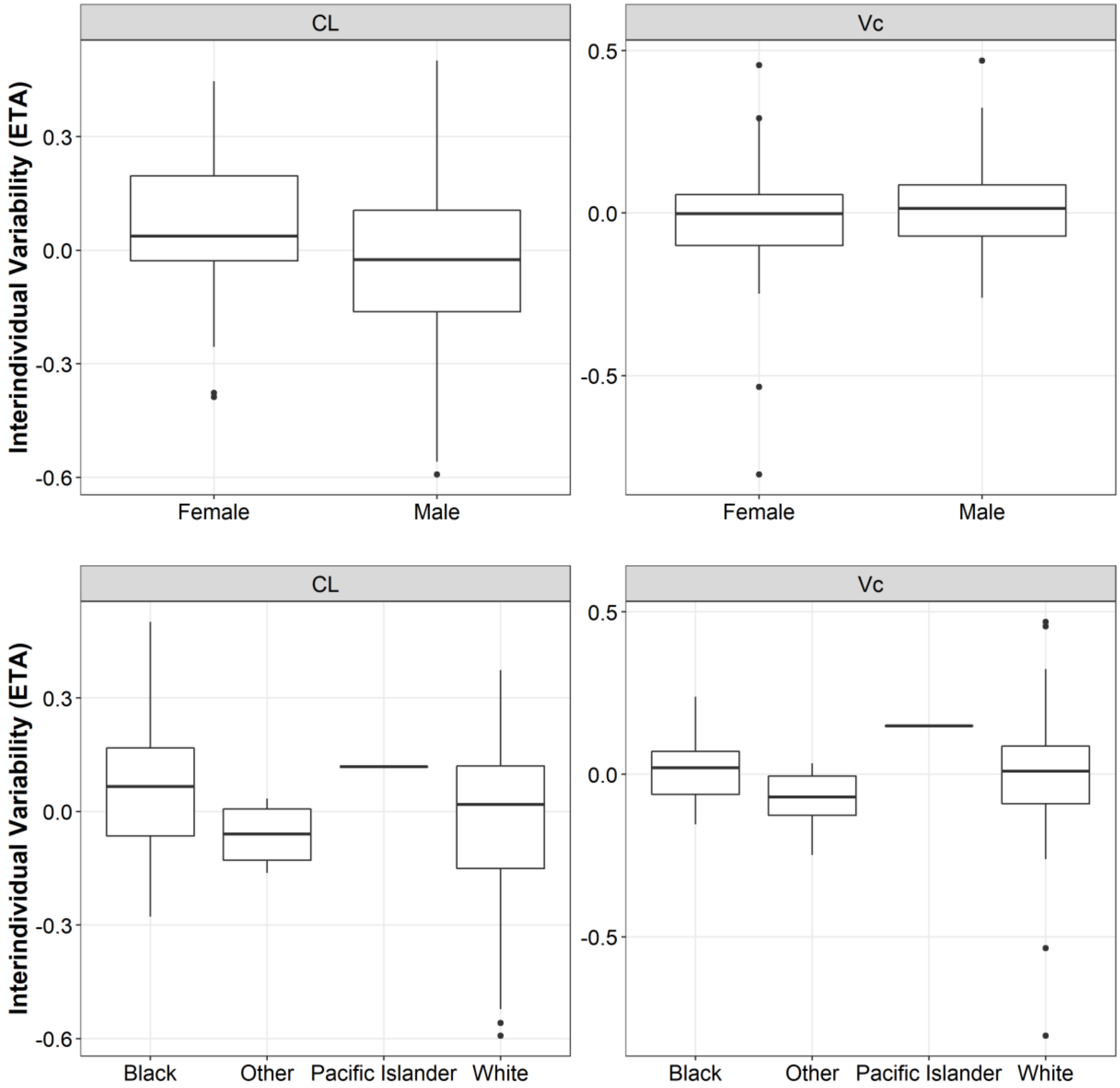

Note: CL, clearance; ETA, patient-specific interindividual variability estimate; Vc, volume of distribution in the central compartment.

Figure S5. Characteristics of the simulated pediatric population

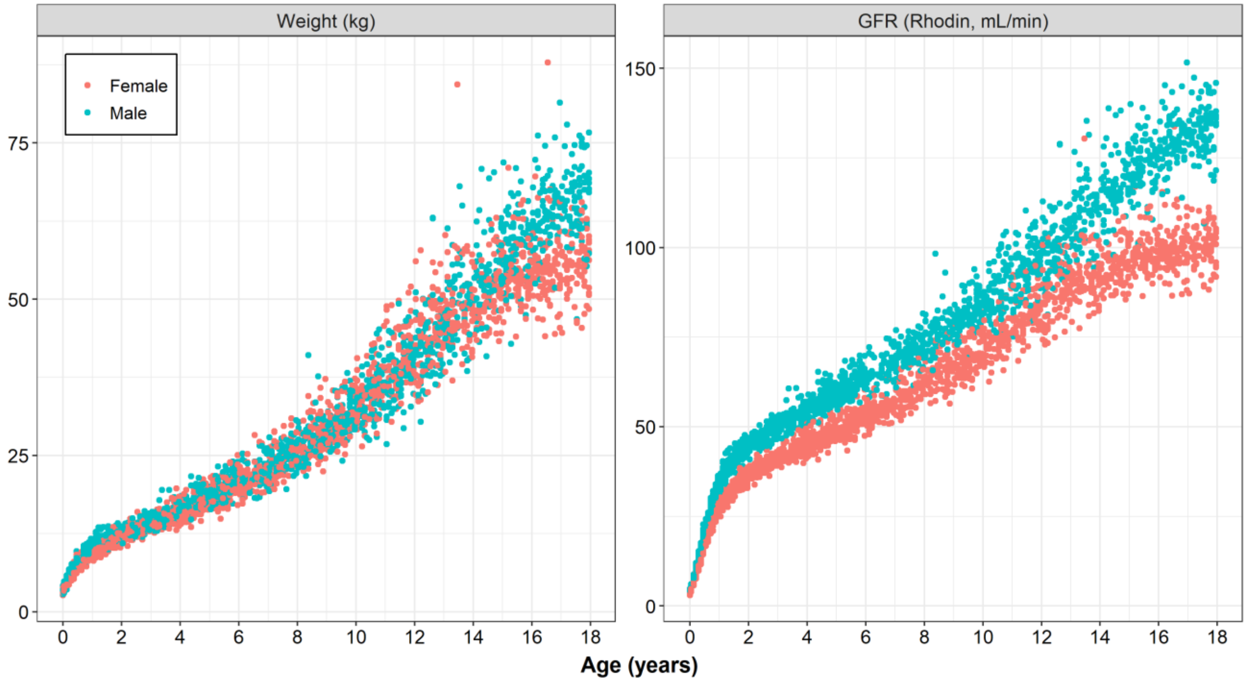

Note: GFR, glomerular filtration rate.

Figure S6. Distributions of predicted ceftobiprole AUC_{0-24} by age group for the optimized dosing regimen in renally impaired patients

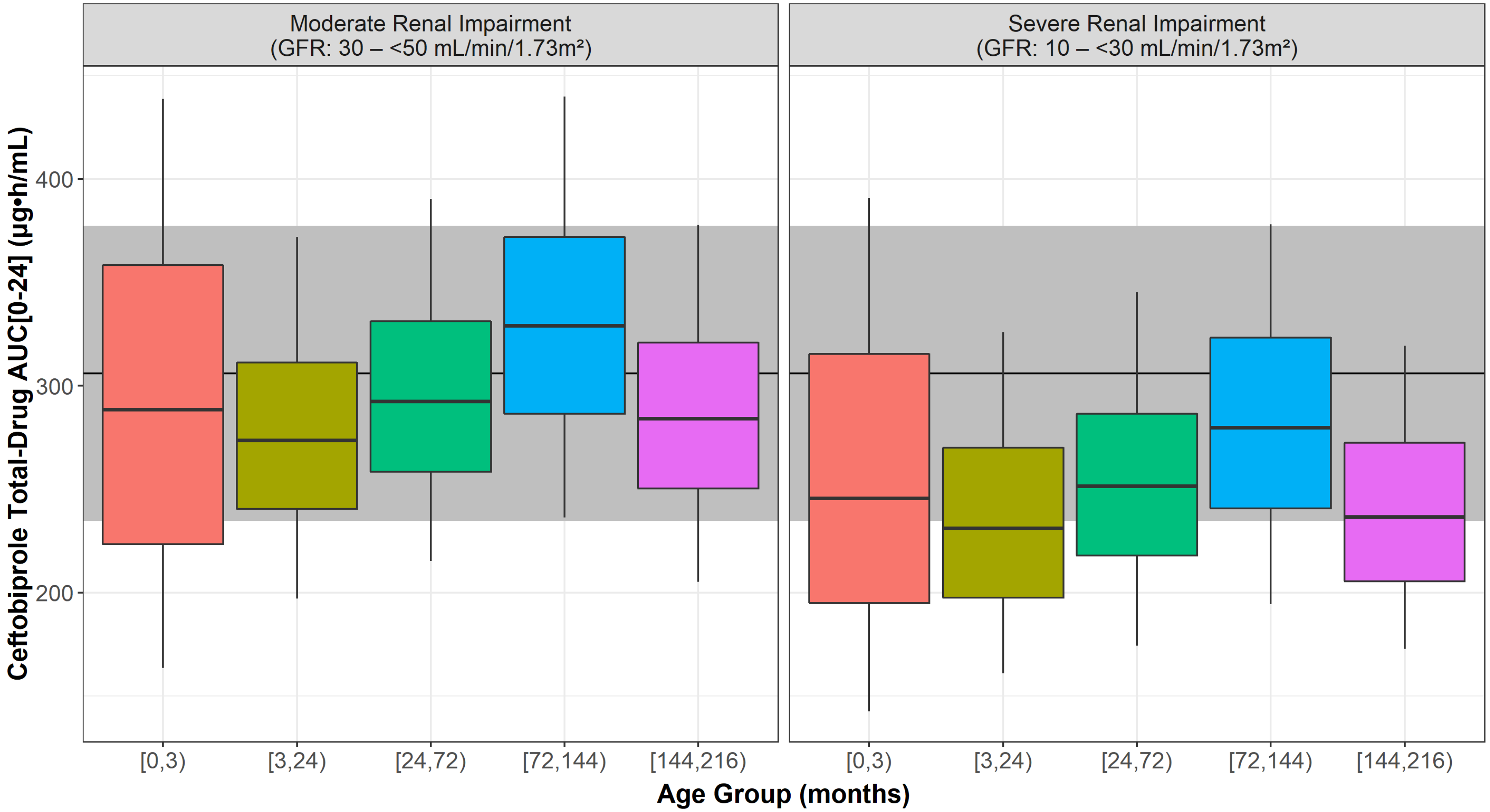

Note: AUC[0-24], area under the plasma concentration-time curve from time zero to 24 hours; GFR, glomerular filtration rate. Dosing regimens provided in **Table 4**. Black line and shaded region is mean ± 2 standard deviations for adults from CSI-1004 (500 mg over 2 hours every 8 hours). Box-and-whisker plots show median, 25th – 75th percentile, and 5th – 95th percentile.

Figure S7. Distributions of predicted ceftobiprole C_{max} by age group for the optimized dosing regimen in renally impaired patients

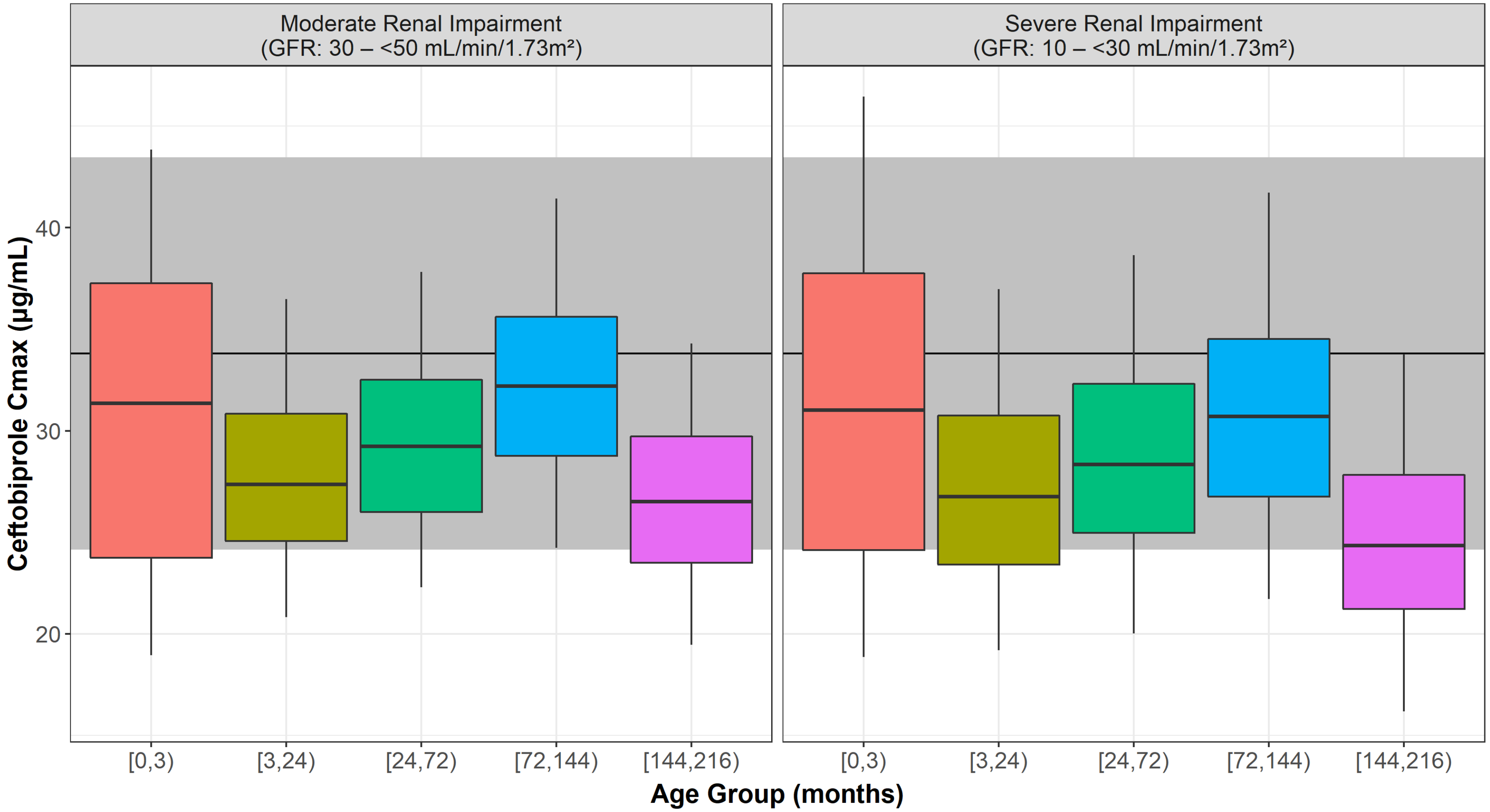

Note: Cmax, maximum plasma concentration; GFR, glomerular filtration rate. Dosing regimens provided in **Table 4**. Black line and shaded region is mean ± 2 standard deviations for adults from CSI-1004 (500 mg over 2 hours every 8 hours). Box-and-whisker plots show median, 25th – 75th percentile, and 5th – 95th percentile.

Table S1. Summary statistics for predicted ceftobiprole AUC_{0-24} and C_{max} by age group for the optimized dosing regimen (with weight adjustment)

Age Group	Dosing regimen	Mean	SD	Median	Min	25th	75th	Max
**AUC_{0-24} (µg•h/mL)**								
Birth to <3 months	15 mg/kg q12h[a]	271	61.1	263	122	228	310	563
3 months to <2 years	15 mg/kg q8h	266	68.0	260	117	217	304	583
2 to <6 years	15 mg/kg q8h	256	55.9	254	131	217	290	453
6 to <12 years	15 mg/kg q8h	297	68.0	289	116	249	338	623
12 to <18 years	15 mg/kg q8h	240	61.0	231	105	195	280	491
**C_{max} (µg/mL)**								
Birth to <3 months	15 mg/kg q12h[a]	29.8	6.53	29.5	14.3	24.5	34.3	51.0
3 months to <2 years	15 mg/kg q8h	28.8	5.17	28.2	15.2	25.2	31.7	51.8
2 to <6 years	15 mg/kg q8h	28.8	4.72	28.4	17.1	25.5	31.7	46.0
6 to <12 years	15 mg/kg q8h	31.9	5.53	31.5	13.7	27.8	35.4	56.0
12 to <18 years	15 mg/kg q8h	24.3	5.21	23.8	12.1	20.6	27.6	43.6

Note: AUC_{0-24}, area under the plasma concentration-time curve from time zero to 24 hours; C_{max}, maximum plasma concentration; Max, maximum; Min, minimum; q8h, every 8 hours; q12h, every 12 hours; SD, standard deviation. All doses infused over 2 hours. The maximum allowable dose to be administered was 500 mg regardless of patient’s weight.

a. Patients with a body weight <4 kg given 10 mg/kg q12h as a 2-hour infusion.

Table S2. Predicted PK-PD target attainment by age group for the optimized dosing regimen in patients with renal impairment

Target (%*f*T>MIC)	Birth to <3 months	3 months to <2 years	2 to <6 years	6 to <12 years	12 to <18 years
	**Moderate renal impairment (GFR: 30 to <50 mL/min/1.73 m^2)**				
	**15 mg/kg q12h[a]**	**10 mg/kg q12h**	**10 mg/kg q12h**	**10 mg/kg q12h**	**7.5 mg/kg q12h**
30%	99.9	100	100	100	100
40%	99.4	99.8	100	99.9	100
50%	95.8	98.7	99.7	99.4	99.9
60%	87.3	94.7	96.9	98.2	99.5
	**Severe renal impairment (GFR: 10 to <30 mL/min/1.73 m^2)**				
	**15 mg/kg q24h[a]**	**10 mg/kg q24h**	**10 mg/kg q24h**	**10 mg/kg q24h**	**7.5 mg/kg q24h**
30%	99.9	99.9	100	100	100
40%	94.6	98.1	99.6	99.8	99.9
50%	82.5	89.2	92.9	97.0	97.5
60%	68.8	73.6	81.6	89.8	88.3

Note: GFR, glomerular filtration rate; %fT>MIC, percentage of time free-drug concentrations are above the minimum inhibitory concentration; q12h, every 12 hours; q24h, every 24 hours. All regimens administered as a 2-hour infusion with a maximum allowable dose of 500 mg regardless of patient's weight.

a. Patients with a body weight <4 kg given 10 mg/kg instead of 15 mg/kg.
